# Potent Anti-HIV Chemokine Analogs Direct Post-Endocytic Sorting of CCR5

**DOI:** 10.1371/journal.pone.0125396

**Published:** 2015-04-29

**Authors:** Claudia Bönsch, Mihaela Munteanu, Irène Rossitto-Borlat, Alexandre Fürstenberg, Oliver Hartley

**Affiliations:** 1 Department of Pathology and Immunology, Faculty of Medicine, University of Geneva. Geneva, Switzerland; 2 Department of Human Protein Sciences, Faculty of Medicine, University of Geneva, Geneva, Switzerland; University of San Francisco, UNITED STATES

## Abstract

G protein-coupled receptors (GPCRs) are desensitized and internalized following activation. They are then subjected to post-endocytic sorting (degradation, slow recycling or fast recycling). The majority of research on post-endocytic sorting has focused on the role of sequence-encoded address structures on receptors. This study focuses on trafficking of CCR5, a GPCR chemokine receptor and the principal entry coreceptor for HIV. Using Chinese Hamster Ovary cells stably expressing CCR5 we show that two different anti-HIV chemokine analogs, PSC-RANTES and 5P14-RANTES, direct receptor trafficking into two distinct subcellular compartments: the trans-Golgi network and the endosome recycling compartment, respectively. Our results indicate that a likely mechanism for ligand-directed sorting of CCR5 involves capacity of the chemokine analogs to elicit the formation of durable complexes of CCR5 and arrestin2 (beta-arrestin-1), with PSC-RANTES eliciting durable association in contrast to 5P14-RANTES, which elicits only transient association.

## Introduction

G protein-coupled receptors (GPCRs) are a highly versatile superfamily of cellular transducers whose physiological roles include the detection of light and odor as well as responses to a diverse range of signaling molecules. They comprise an estimated 4% of the coded genome [1] and are the targets of more than 30% of licensed medicines [2]. The physiological function of GPCRs depends on their capacity to undergo desensitization following activation and signaling. Desensitization is orchestrated by intracellular arrestin proteins [3, 4], which (i) sterically block G protein signaling by binding to the cytosolic face of the receptor, (ii) act as scaffolds for the recruitment of the endocytic machinery, removing activated receptors from the cell surface, and (iii) elicit intracellular signaling through G protein-independent pathways.

Following endocytosis, the GPCR superfamily can be divided into receptors that are targeted for degradation and those that are recycled to the cell surface in a resensitized form [5, 6]. Recycled receptors can be further subdivided into those that are recycled rapidly and those that are recycled more slowly [3, 5, 6]. The post-endocytic sorting process is governed by interactions of GPCRs with key intracellular proteins, including arrestins, with most research focusing on the presence or absence of sequence-encoded interaction domains for these proteins on the receptor [3, 5, 6]. Only a few examples of ligand-driven post-endocytic sorting of GPCRs have been described previously [7–9].

The chemokine receptor CCR5 is a member of the G protein-coupled receptor (GPCR) superfamily. Although its main physiological role is the recruitment of effector cells to sites of inflammation [10], CCR5 is also the principal coreceptor used by HIV to enter and infect target cells, and is therefore an attractive target for HIV prevention and therapy [11]. Since the discovery that the natural ligands of CCR5, MIP-1α/CCL3, MIP-1β/CCL4 and RANTES/CCL5 exhibit anti-HIV activity [12, 13], a number of analogs with significantly increased potency have been described [14]. Among these, AOP-RANTES [15] and PSC-RANTES [16, 17], were shown to be CCR5 superagonists [18, 19] that owe their potent inhibitory activity to their capacity to induce profound and long-term intracellular sequestration of CCR5 [20–22]. A second group of analogs discovered using a modified phage display approach [23] included analogs such as 5P14-RANTES that induce receptor sequestration in the absence of G protein signaling, and analogs such as 5P12-RANTES that elicit neither receptor sequestration nor G protein signaling [24]. It has been suggested that the different capacities of these ligands to achieve intracellular CCR5 sequestration is likely to be due to differences in the conformations they elicit CCR5 to adopt, which in turn modulate interactions between CCR5 and the cellular desensitization machinery [25].

CCR5 belongs to the group of GPCRs that is recycled after desensitization [20, 21]. CCR5 internalized by native ligands is transported to the trans-Golgi network (TGN) via the endosome recycling compartment (ERC) [22], from where it cycles to and from the cell surface until the resensitization process is complete [21, 22]. AOP-RANTES and PSC-RANTES direct CCR5 through the same trafficking pathway as the native ligands [22], albeit with sequestration of longer duration than that induced by the natural ligands [16, 20]. The trafficking pathway taken by CCR5 internalized by 5P14-RANTES has not yet been investigated.

Here we compared the intracellular trafficking pathway taken by CCR5 internalized by 5P14-RANTES with that of CCR5 internalized by PSC-RANTES. We demonstrate that the fate of internalized CCR5 can be determined by the ligand that engaged it, and our results indicate that that the duration of ligand-induced receptor-arrestin association is likely to play a key role in the sorting mechanism.

## Materials and Methods

### Cell Lines

Chinese Hamster Ovary (CHO) cells expressing CCR5 (CHO-CCR5) used in this study have been described previously [22, 24]. Stable CHO-CCR5 lines expressing arrestin2-GFP (plasmid p-arrestin2-GFP [26], kindly provided by Jeff Benovic) were generated as described previously [27, 28].

### Chemokines

Chemokines and chemokine analogs used in this study were prepared by total chemical synthesis as described previously [16, 24].

### Antibodies

A full list of antibodies used in this study is provided in Table 1. For live microscopy, the anti-CCR5 monoclonal antibody Hek/1/85a (AB_369016, Serotec) was rhodamine-labeled using amine-reactive *N*-hydroxy-succinimidyl-Rhodamine (Pierce) according to the manufacturer’s instructions.

**Table 1:** Antibodies used in this study.

Antibody	Method (Figures in main article)	Reference*
**Primary antibodies**		
anti-CCR5, 3A9, mouse	Immunofluorescence on fixed cells (Fig 1 and Fig 3)	AB_2072548
anti-CCR5, Hek / 1 / 85a, rat	Immunoprecipitation (Fig 4); live cell immunofluorescence (Fig 5)	AB_369016
anti-CCR5, R22/7, mouse	Western blot (Fig 2)	AB_626823
anti-TGN38, antisera 1479, rabbit	Immunofluorescence on fixed cells (Fig 1 and Fig 3)	B.A. Eipper (Milgram et al. (1997) *J Cell Sci* **110** p695)
anti-Rab11, rabbit	Immunofluorescence on fixed cells (Fig 1 and Fig 3)	AB_87868
anti-GFP, mouse	Western blot (Fig 4)	AB_390913
anti-arrestin2, rabbit	Western blot (Fig 4)	AB_722898
anti-CCR5pSer349, E11/19, mouse	Immunofluorescence on fixed cells (Fig 6)	AB_567390
**Secondary antibodies**		
Alexa-Fluor488-donkey anti-mouse	Immunofluorescence on fixed cells (Fig 1, Fig 3 and Fig 6)	AB_10049285
rhodamine-donkey anti-rabbit	Immunofluorescence on fixed cells (Fig 1 and Fig 3)	AB_2340588
HRP-goat anti-mouse	Western blot (Fig 4)	DAKO Cytomation P0447
HRP-goat anti-rabbit	Western blot (Fig 4)	DAKO Cytomation P0448
HRP-goat anti-rat IgG	Western blot (Fig 4)	AB_11214444
HRP-rabbit anti-mouse	Western blot (Fig 4)	DAKO Cytomation Z0259

*where possible, stable public identifiers from the Antibody Registry (www.antibodyregistry.org) are provided; HRP; Horseradish peroxidase.

### Immunofluorescence microscopy

Experiments were performed as described previously [22], except that in certain experiments an acid wash step (150 mM NaCl, 75 mM Glycine-HCl, pH 2.5) was used to remove antibody remaining at the cell surface prior to fixing and revelation. Image analysis was performed using Zeiss LSM Image Browser Version 4.2.0121 with ZEN software 2011.

### G protein signaling assay

G_i/o_ protein-dependent signaling activity of chemokines was determined by measuring reduction in forskolin-stimulated cAMP levels using a commercially available luminescence-based assay (cAMP eXpress kit, DiscoveRx) according to the manufacturer’s instructions. IC_50_ values were determined from dose-response curves fitted using Prism software (GraphPad). Where required, CHO-CCR5 cells were pre-incubated (100 ng/mL, overnight) with Pertussis toxin (Sigma) prior to assay.

### CCR5-arrestin2 co-immunoprecipitation assay

CHO-CCR5 cells stably expressing arrestin2-GFP [27, 28] growing on 150 mm petri dishes were treated with chemokines (50 min, 100 nM) then washed extensively in PBS. Cells were further incubated 60 min with 1.25 mM dithiobis [succinimidylpropionate] cell-permeable crosslinker (Pierce) then quenched by incubation with 20 mM Tris (pH 7.5, 20 min), washed twice and lysed (25 mM Tris, 150 mM NaCl, 1 mM EDTA, 1% NP40, 5% glycerol, supplemented with protease inhibitor cocktail (Roche)). Lysates were pre-cleared by incubating 2 h with Protein G Sepharose beads (GE healthcare) coated with rabbit anti-mouse IgG, and CCR5 capture was carried out using Protein G Mag Sepharose beads (GE Healthcare) preincubated with rat anti-CCR5 mAb (20 μg/sample, AB_369016). Sample elution was carried out according to the manufacturer’s instructions, with crosslinks cleaved by incubation with 50 mM DTT for 120 min at 37°C. Immunoprecipitates were subjected to SDS-PAGE (4–12% BisTris precast gels, Invitrogen), and Western Blot was carried out with arrestin-GFP detected either with a mouse anti-GFP antibody (0.4 μg/mL, AB_390913) or a polyclonal rabbit anti arrestin2 antibody (1 μg/mL, AB_722898). A monoclonal mouse anti-CCR5 antibody (0.3 μg/mL, AB_626823) was used to control total levels of captured CCR5 in immunoprecipitates.

### Live cell fluorescence microscopy

CHO-CCR5 cells stably expressing arrestin2-GFP [27, 28] growing on glass petri dishes coated with fibronectin (Calbiochem, 5μg/mL) were subjected to laser scanning confocal microscopy using a Nikon A1r confocal microscope with a 488 nm laser and with a 60x CFI plan apo VC 1.4 objective lens. After 3 min acquisition, chemokines (100 nM) were added, followed by a further 60 min acquisition. Image analysis was performed using NIS Elements AR software (Nikon Corporation).

## Results

### Ligand-directed post-endocytic sorting of CCR5

**Fig 1:**
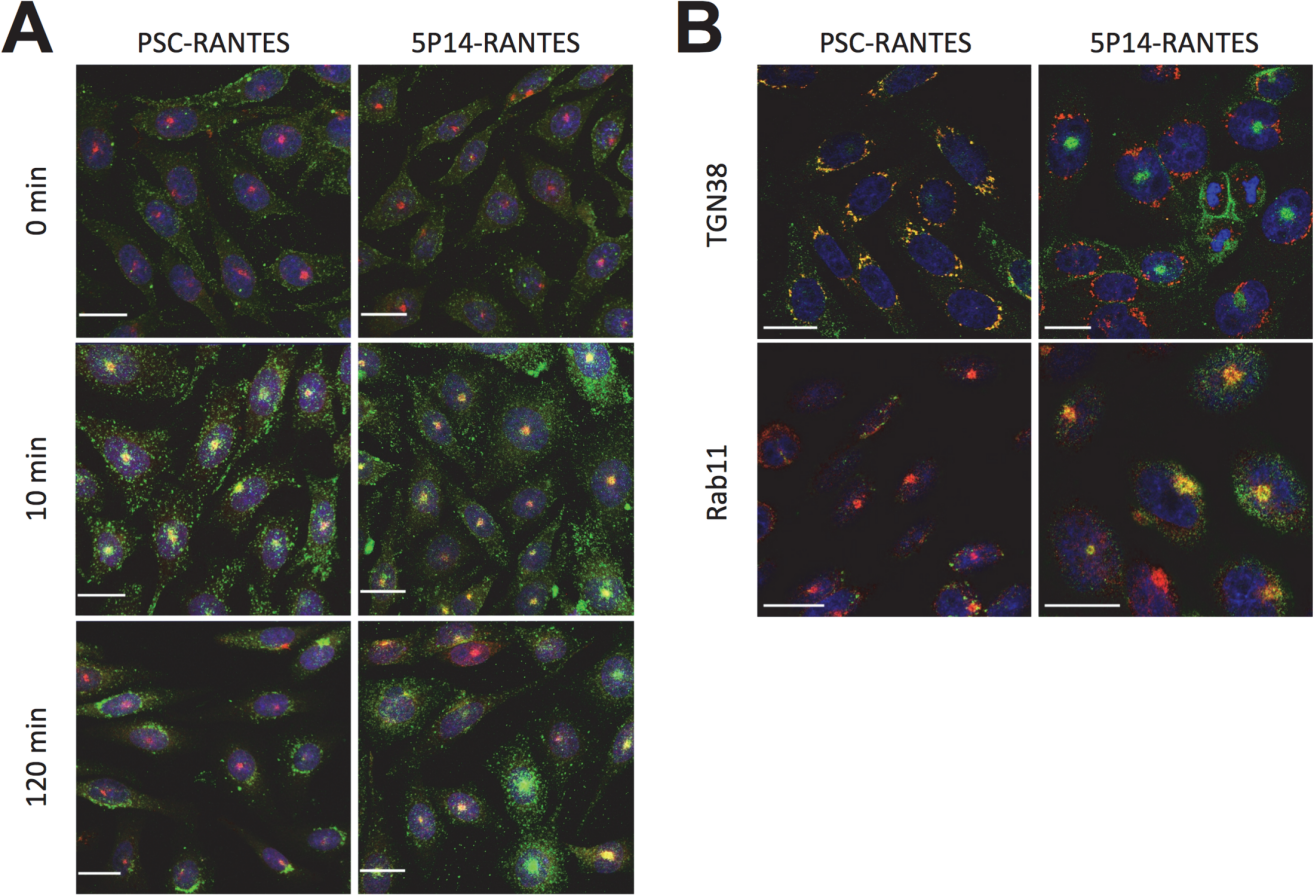
Ligand-directed post-endocytic sorting of CCR5. **A.** CHO CCR5 cells growing on coverslips were pre-treated (60 min, 4°C) with 100 nM chemokines and anti-CCR5 antibody 3A9 (green), then washed and incubated at 37°C for the indicated times. Cells were then acid washed to remove cell surface antibody, then fixed, permeabilized, and labeled for the ERC marker Rab11 (red) and DAPI (blue, nuclear staining) prior to analysis by confocal scanning microscopy are shown, scale bar = 20 μm. Throughout the experiment the ERC marker (red) remains in a discrete supranuclear spot that is visble in the center of the nuclei (blue) in the maximum intensity projections. Initially, CCR5 (green) is localized at the cell surface, but after 10 min incubation with either ligand it translocates to colocalize with the ERC marker. After 120 min incubation, CCR5 on cells treated with PSC-RANTES subsequently relocates from the ERC to accumulate in a perinuclear site (ring-shaped staining around the nuclei), while CCR5 in cells treated with 5P14-RANTES remains colocalized with the ERC marker. **B.** Individual Z-slice images from an identical experiment in which cells incubated with the indicated chemokines for 180 min were also labeled for either the ERC marker, Rab11 or the TGN marker, TGN38 (red). Slices in which the marked compartment is most abundant (through the middle of the nucleus for TGN, just above the nucleus for ERC) were chosen. While in cells treated with PSC-RANTES for 180 min, CCR5 colocalizes with TGN38 and does not colocalize with Rab11, in cells treated with 5P14-RANTES for 180 CCR5 colocalizes with Rab11 and does not colocalize with TGN38 scale bar = 20 μm.

To compare intracellular trafficking of CCR5 internalized by 5P14-RANTES with that of CCR5 internalized by PSC-RANTES, we used an anti-CCR5 antibody together with compartment-specific antibodies (anti-Rab11 for the ERC, anti-TGN38 for the TGN) to perform pulse-chase colocalization immunofluorescence microscopy experiments essentially as described previously [22] (Fig 1A). As expected from previous work [22], CCR5 internalized by PSC-RANTES initially accumulates in a discrete supranuclear Rab11-positive patch that corresponds to the ERC [22, 29], subsequently relocating to a perinuclear region consistent with the TGN [22]. In striking contrast, CCR5 internalized by 5P14-RANTES accumulates in the supranuclear ERC patch with no subsequent migration to the perinuclear compartment. These differences in subcellular localization are clearly apparent in individual z-slice images obtained in a follow-up experiment in which chemokine-treated cells were stained for CCR5 plus either Rab11 (ERC) or TGN38 (TGN) (Fig 1B).

### Independence from G protein signaling

Since 5P14-RANTES was previously shown to internalize CCR5 in the absence of G protein signaling [24], we first tested the hypothesis that differences in G protein signaling might drive ligand-directed post-endocytic sorting, with G protein signaling providing the additional cue necessary to traffic CCR5 out of the ERC and into the TGN.

**Fig 2:**
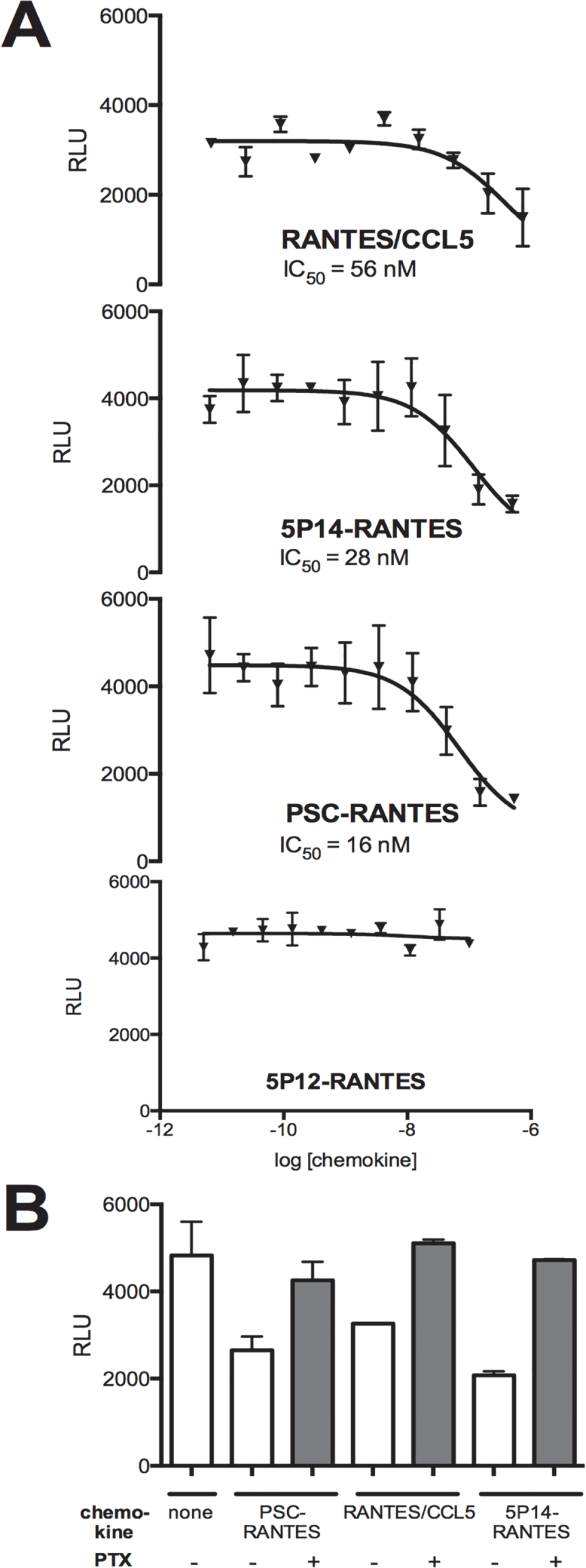
G protein signaling of chemokine analogs on CHO-CCR5 cells. **A.** G_i/o_ protein signaling activity of chemokines at the indicated doses was determined by measuring reduction in forskolin-stimulated cAMP levels. Results shown (Relative Light Units, RLU) are the mean of duplicate readings, with error bars indicating the range. **B.** G protein signaling activity of chemokines (100 nM) was determined by measuring reduction in forskolin-stimulated cAMP levels. Where indicated CHO-CCR5 cells were pre-incubated (100 ng/mL, overnight) with Pertussis toxin (PTX). Results shown (Relative Light Units, RLU) are the mean of duplicate readings, with error bars indicating the range.

Chemokine receptors couple to G_i/o_ proteins, and upon activation the released α_i/o_ subunits reduce intracellular levels of cAMP through inhibition of adenylate cyclase, while the released βγ subunits activate phospholipase C, eliciting IP_3_-mediated intracellular calcium flux [30]. In previous work, the G protein signaling activity of 5P14-RANTES and PSC-RANTES was determined by measuring calcium flux in both HeLa cells engineered to express CCR5 and in human T cells [24]. No detectable calcium flux signals were obtainable using the CHO-CCR5 cell line used in this study (OH, unpublished results), so we opted instead to measure inhibition of forskolin-stimulated cAMP levels (Fig 2A). We found that RANTES/CCL5 and PSC-RANTES, but not 5P12-RANTES, elicited dose-dependent reduction of cAMP levels, in line with previous observations [19, 24]. Unexpectedly, 5P14-RANTES also elicited dose-dependent reduction of forskolin-stimulated cAMP levels, with similar potency and efficacy to RANTES/CCL5 and PSC-RANTES. It has been established that signaling profiles of GPCR ligands can be modified according to the cellular background used [31], and this is the most likely explanation for the differences in signaling activity determined for 5P14-RANTES in this study compared to that observed previously [24].

**Fig 3:**
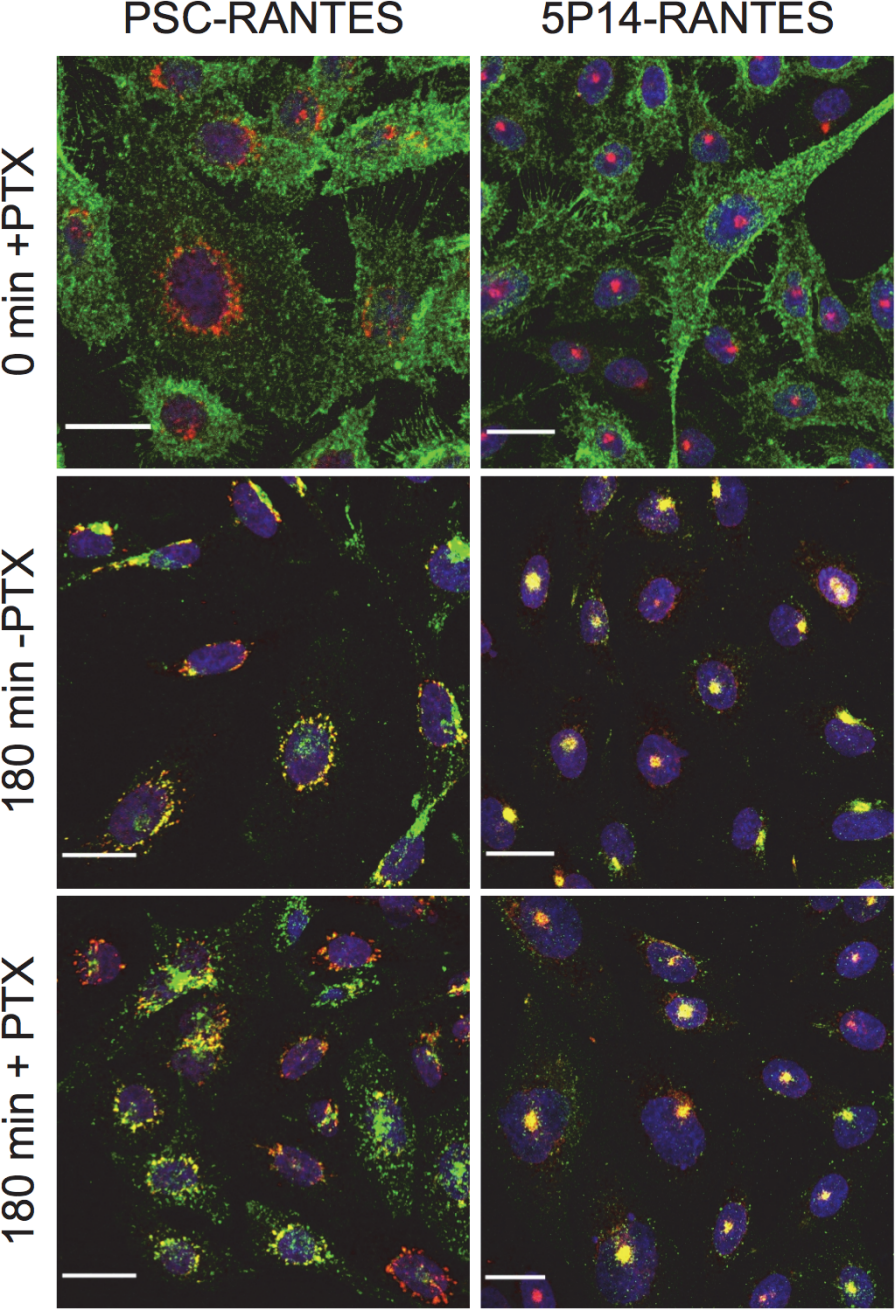
Ligand-directed post-endocytic sorting of CCR5 is not dependent on G protein signaling. CHO CCR5 cells growing on glass coverslips were incubated overnight with Pertussis toxin (100 ng/mL), then washed and stimulated with 100 nM chemokines as indicated in the presence of anti-CCR5 antibody 3A9 (green) at 37°C. Cells were then acid washed (15 min 4°C) to remove cell surface antibody, fixed, permeabilized, and labelled for either the ERC marker Rab11 (5P14-RANTES) or the TGN marker TGN38 (PSC-RANTES) (red). After staining with DAPI nucleic acid stain (blue), cells were analysed by confocal microscopy. Maximum intensity projections are shown, scale bar = 20 μm. Throughout the experiment the ERC and TGN markers (red) remain either in a discrete supranuclear spot (ERC) or at a perinuclear site (ring-shaped staining around the nuclei, TGN). Initially, CCR5 (green) is localized at the cell surface, but after 10 min incubation with either ligand it translocates to the supranuclear spot (visibly colocalizing with the ERC marker used in the cells treated with 5P14-RANTES). After 120 min incubation, CCR5 on cells treated with PSC-RANTES subsequently relocates from the ERC to accumulate in a perinuclear site (ring-shaped staining around the nuclei, visibly colocalizing with the TGN marker), while CCR5 in cells treated with 5P14-RANTES remains colocalized with the ERC marker.

Reduction of cAMP levels elicited by each ligand (100 nM) was fully abrogated by pretreatment with Pertussis toxin (100 ng/mL, overnight), which blocks coupling of GPCRs to Gα_i/o_ proteins (Fig 2B). When CHO-CCR5 cells were pretreated with pertussis toxin under the same conditions prior to incubation with ligands (100 nM, 180 min), no change in either the extent of CCR5 internalization or the subcellular localization of internalized CCR5 was observable (Fig 3). Hence G_i/o_ protein signaling does not provide an additional cue necessary to traffic internalized CCR5 out of the ERC and into the TGN.

### Ligand-Driven Arrestin-CCR5 Association

Since it is known that ligands of a given GPCR can differ in their capacity to elicit receptor-arrestin association, and that arrestins are key orchestrators of GPCR desensitization [3, 4], we next tested the hypothesis that differences in ligand-driven arrestin recruitment are responsible for ligand-directed post-endocytic sorting of CCR5.

**Fig 4:**
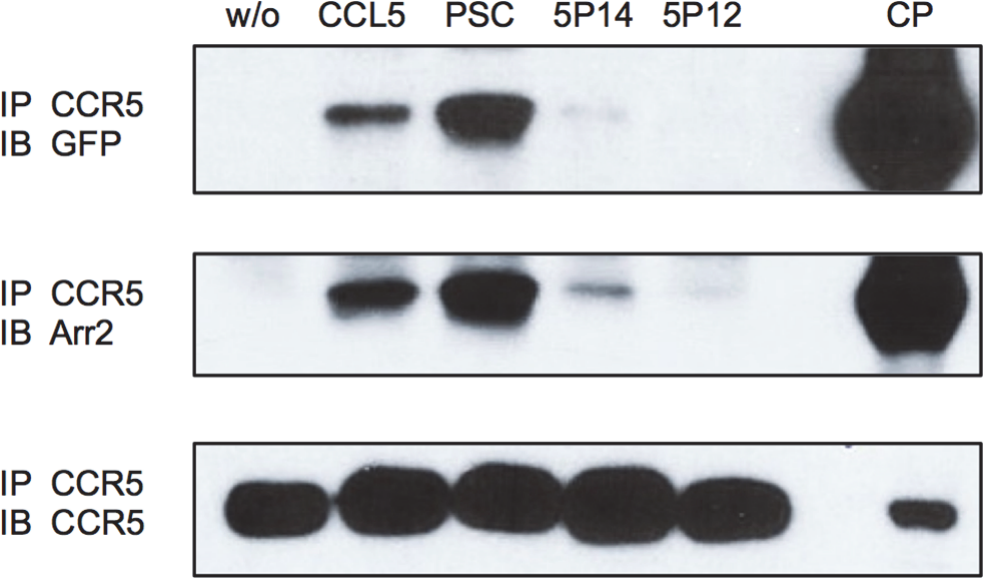
Ligand-elicited recruitment of arrestin2 to CCR5. CHO-CCR5 cells stably transfected with arrestin2-GFP were treated with the indicated chemokines (100 nM, 50 min), washed extensively, crosslinked, washed again and lysed prior to immunoprecipitation with an anti-CCR5 antibody. Immunoprecipitates were subjected to Western blot using antibodies against GFP, arrestin2 and CCR5. CP; total cell pellet control, IP; antibody specificity used for immunoprecipitation, IB; antibody specificity used for immunoblot.

We performed arrestin2-CCR5 co-immunoprecipitation experiments in which CHO-CCR5 cells expressing arrestin2 fused to GFP (arrestin2-GFP) were incubated with ligands prior to lysis and immunoprecipitation using a CCR5-specific antibody. Associated levels of arrestin2 were then detected by Western blot using antibodies specific for either arrestin2 or GFP (Fig 4). As expected [18, 32, 33], RANTES/CCL5 induced recruitment of arrestin2-GFP. PSC-RANTES elicited clearly increased levels of arrestin2-GFP recruitment, and while recruitment of arrestin2-GFP recruitment elicited by 5P14-RANTES was detectable, it was distinctly lower than the level obtained with CCL5. 5P12-RANTES did not elicit any detectable arrestin2-GFP recruitment.

**Fig 5:**
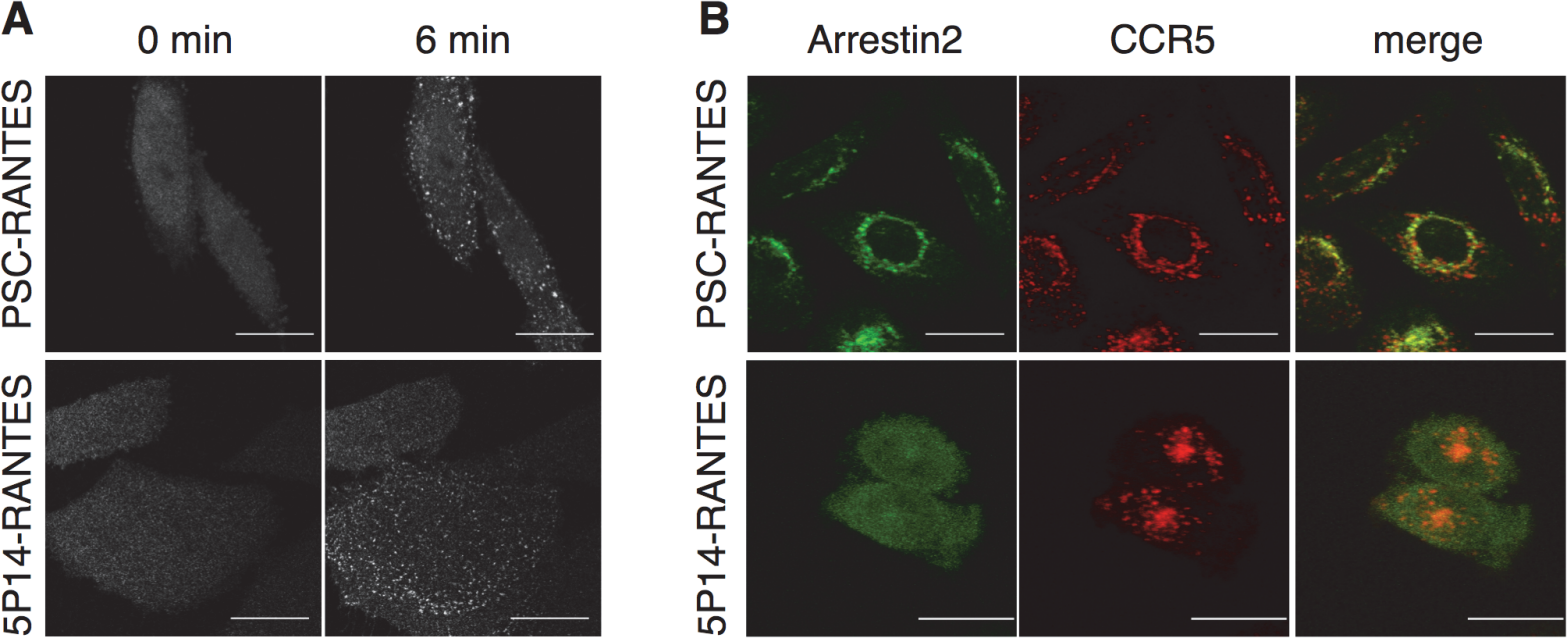
Spatial and temporal resolution of arrestin2-CCR5 association. **A** CHO-CCR5 cells stably transfected with arrestin2-GFP were treated with chemokine analogs (100 nM) as indicated and the redistribution of arrestin2-GFP was followed by live fluorescence microscopy. Images captured prior to (0 min) and after ligand treatment (6 min) are shown. **B** CHO-CCR5 cells stably transfected with arrestin2-GFP (green), preincubated with rhodamine-labeled anti-CCR5 antibody (red), were washed and then incubated 90 min at 37°C with chemokine analogs (100 nM) prior to image capture. Maximal intensity projections are shown. Scale bar = 20 μm.

We next set out to investigate the localization and duration of arrestin2 association with CCR5 following incubation with the different ligands (Fig 5). Live immunofluorescence microscopy was used to monitor the localization of arrestin2-GFP in CHO-CCR5 cells following addition of either PSC-RANTES or 5P14-RANTES (100 nM). As seen in previous work [27, 28], treatment with both PSC-RANTES and 5P14-RANTES led to rapid relocalization of arrestin2-GFP from a diffuse cytosolic distribution to a punctate pattern located close to the plasma membrane (Fig 5A).

In a second experiment, CHO-CCR5 cells stably expressing arrestin-GFP were pre-labeled at 4°C with a rhodamine-conjugated anti-CCR5 antibody, then washed and incubated with either PSC-RANTES or 5P14-RANTES for 60 min prior to image capture (Fig 5B). As expected, CCR5 was internalized in PSC-RANTES-treated cells, accumulating in the perinuclear region. Arrestin2-GFP clearly colocalized with sequestered CCR5 at this site. In striking contrast, while 5P14-RANTES treatment led to sequestration of CCR5 in the supranuclear ERC patch as expected, the bulk of arrestin2-GFP did not colocalize with internalized CCR5, remaining diffusely spread through the cytoplasm.

### GRK-mediated CCR5 phosphorylation

**Fig 6:**
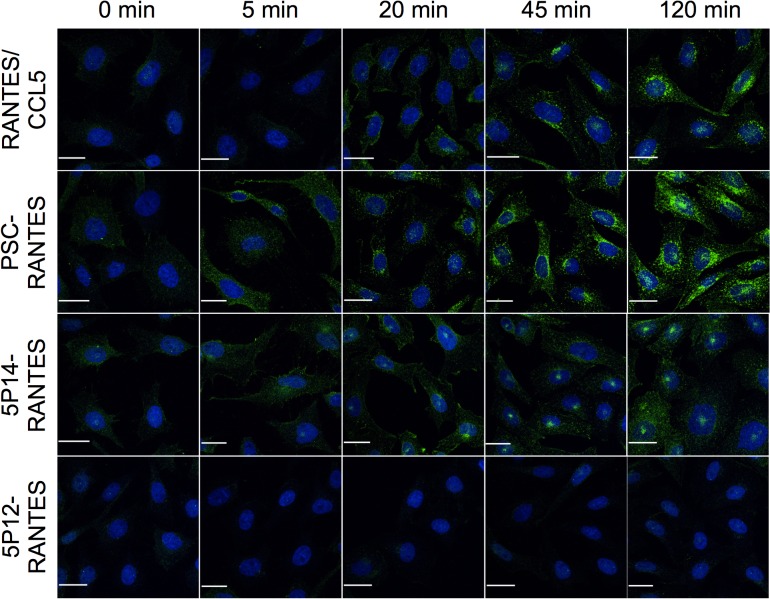
Ligand-induced GRK phosphorylation of CCR5. CHO-CCR5 cells growing on coverslips were treated with the indicated chemokines (100 nM) for the indicated times, then washed, fixed, permeabilized and labeled with a monoclonal antibody specific for CCR5 phosphorylated on Serine 349 (green) and DAPI nucleic acid stain (blue). Maximal intensity projections are shown. Scale bar = 20 μm.

Phosphorylation of serine and threonine residues in the cytoplasmic C-terminal tail of GPCRs by G protein-coupled receptor kinases (GRKs), is known to facilitate arrestin association [3, 6, 34], and C-terminal phosphorylation of CCR5 has been shown to play a key role in the desensitization process [35]. In order to investigate a potential role for GRK phosphorylation in ligand-directed post-endocytic sorting of CCR5, we used a monoclonal antibody specific for CCR5 phosphorylated at Ser349, one of several C-terminal serine residues previously shown to be targeted for GRK phosphorylation [35], in an immunofluorescence microscopy experiment to assess the extent and localization of phospho-Ser349-CCR5 in CHO-CCR5 cells following ligand treatment at time intervals up to 120 min (Fig 6). As expected from previous work [35], RANTES/CCL5 elicits phosphorylation of CCR5 on Ser 349. Accumulating phospho-Ser349-CCR5 is predominantly localized in the perinuclear region, consistent with the TGN site at which the bulk of total internalized CCR5 accumulates [22]. PSC-RANTES elicits more robust phosphorylation at Ser349, with the bulk of staining again visible in the perinuclear region of the cells. 5P14-RANTES also clearly elicits Ser349 phosphorylation of CCR5, with time-dependent signal accumulation comparable to that of the other two ligands. Here again, the phospho-Ser349-CCR5 accumulates in the same location as the bulk of CCR5 internalized by 5P14-RANTES: the ERC region located above the nucleus. 5P12-RANTES did not elicit any detectable phosphorylation at Ser349.

## Discussion

In this study we show that CCR5 internalized by two different ligands, 5P14-RANTES and PSC-RANTES, follows two clearly different trafficking itineraries, with PSC-RANTES-internalized CCR5 trafficking through the ERC to the TGN, as observed previously [22], while 5P14-RANTES-internalized CCR5 is retained in the ERC (Fig 1). This finding is of importance because although post-endocytic sorting guided by interaction domains on the cytosolic surface of GPCRs [3, 5, 6] is well established, and has been documented for CCR5 [36], only a few examples of post-endocytic sorting of GPCRs have been reported [7–9]. Furthermore, these examples concern (i) opioid [7, 9] and adrenergic [8] receptors rather then chemokine receptors, and (ii) sorting between the recycling and degradative pathways, rather than between TGN and ERC.

Since it was previously shown that unlike PSC-RANTES, 5P14-RANTES does not elicit G protein-mediated CCR5 signaling either in transfected HeLa cells or in primary T cells [24], our first hypothesis was that signaling differences would be the explanation for the ligand-directed post-endocytic sorting observed in CHO-CCR5 cells. This is unlikely to be the explanation, however. Firstly, we found that in 5P14-RANTES and PSC-RANTES have comparable G_i/o_ protein signaling activity in CHO-CCR5 cells (Fig 2), and secondly, full abrogation of G_i/o_ protein signaling activity via CCR5 using pertussis toxin did not affect that capacity of either ligands to either (i) elicit receptor endocytosis or (ii) direct internalized receptors to the two different intracellular locations (Fig 3). We cannot exclude the possibility that some other form of G protein signaling comes into play in CHO-CCR5 cells, since promiscuous coupling of GPCRs to more than one class of G protein is a known phenomenon [37], particularly when receptors are expressed a non-physiological background. However, since neither calcium signaling (elicited by G_q_ proteins) nor increases in intracellular cAMP levels (elicited by G_s_ proteins) were detectable, we can rule out promiscuous coupling with G proteins from either of these classes.

Instead, our results indicate that the duration of arrestin recruitment to CCR5 is likely to be key to directing post-endocytic trafficking. PSC-RANTES elicits robust and long-duration of recruitment of arrestin2 to CCR5 (Fig 4 and Fig 5B). In contrast 5P14-RANTES-mediated arrestin2 recruitment appears to be transient in nature, (Fig 5), which would provide an explanation for the weak signal obtained in the co-immunoprecipitation assay (Fig 4), where material was harvested after 50 min ligand exposure.

A post-endocytic sorting mechanism for CCR5 driven by the duration of arrestin-receptor association would seem plausible, since it has been established that members of the GPCR superfamily can be divided into two classes based on their duration of interaction with arrestins during desensitization, which influences the speed by which receptors are recycled following endocytosis [3, 5, 6, 38].

The extent and the quality of arrestin-GPCR interaction is strongly influenced by the activity of G protein-coupled receptor kinases (GRKs) which recognize and phosphorylate ligand-activated GPCRs [3, 4]. Using an antibody specific for CCR5 phosphorylated on Ser349, one of 4 serine residues known to be targets of GRK, we showed that all three ligands capable of eliciting CCR5 internalization (RANTES/CCL5, PSC-RANTES and 5P14-RANTES) also elicit phosphorylation on Ser349 (Fig 6), with PSC-RANTES, which elicits the highest level of arrestin2-GFP recruitment (Fig 4), generating the strongest signal. While it was interesting to note that for each ligand, the site of accumulation of phosphoSer349-CCR5 is consistent with the site of bulk CCR5 accumulation (TGN for RANTES/CCL5 and PSC-RANTES, ERC for 5P14-RANTES), this experiment did not provide evidence that either the extent or the kinetics of GRK phosphorylation of Ser349 is responsible for modulating the duration of ligand-induced arrestin recruitment to CCR5. GRKs may nonetheless play a key role in this phenomenon, however. Evidence is emerging that different ligands of the same GPCR can elicit different patterns of GRK phosphorylation, with the resulting phosphorylation ‘barcodes’ playing a potentially important role in mediating interaction with arrestins [39]. It is conceivable that while both 5P14-RANTES and PSC-RANTES are capable of eliciting Ser349 phosphorylation, they differ in their capacity to elicit GRK phosphorylation at the other sites, and that the resulting ‘barcode’ differences affect the duration of CCR5-arrestin recruitment.

Other post-translational modifications may also contribute to the trafficking mechanism, for example it has been shown that the trafficking itinerary of another chemokine receptor, CCR7, is affected by the extent to which it is ubiquitnylated [40]. Finally, a recent study of the mu-opioid receptor [9] has shown that ligands can influence post-endocytic sorting by differentially eliciting recruitment of arrestin2 versus arrestin3. Our recent observation that while both PSC-RANTES and 5P14-RANTES trigger intracellular clustering arrestin2 in CHO-CCR5 cells, only PSC-RANTES triggers clustering of arrestin3 [27, 28], suggesting that the capacity of the ligands to elicit recruitment of arrestin3 may be an additional mechanism affecting post-endocytic sorting of CCR5.

**Fig 7:**
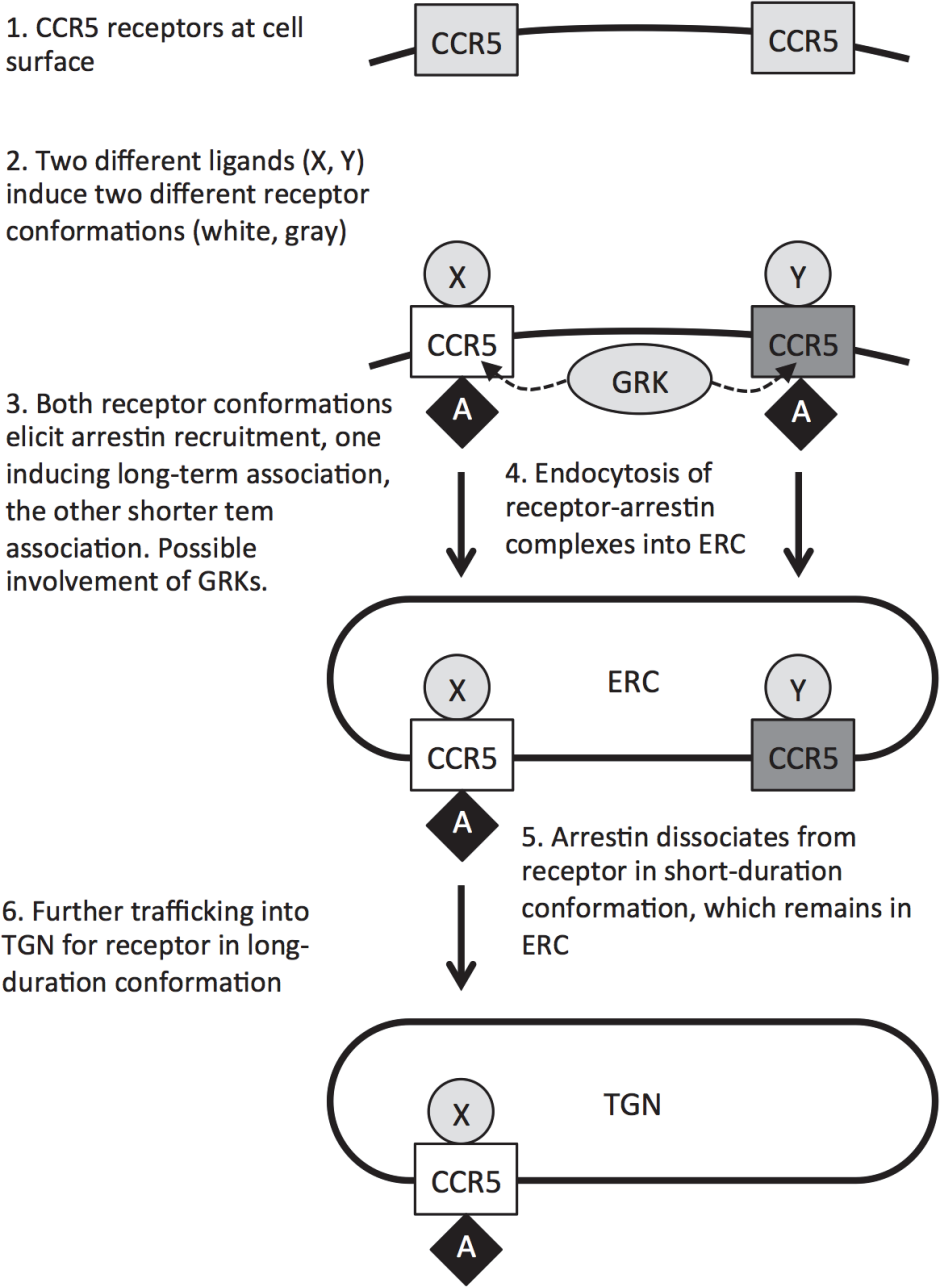
A model for ligand-directed post-endocytic sorting of CCR5.

GPCRs are highly flexible proteins whose conformations are modulated by external ligands and sensed by cytosolic binding partners, which include the G proteins themselves and arrestins [3, 4]. Biased ligands, which favorize either G protein signaling or arrestin recruitment by inducing the receptor to adopt different conformations, have been identified for growing number of GPCRs [34, 41], and it has been proposed that certain chemokine analogs are biased ligands of CCR5 [25]. A simple model (Fig 7) would involve PSC-RANTES and 5P14-RANTES inducing two different CCR5 conformations, both capable of eliciting initial arrestin recruitment, but differing in their capacity to elicit longer duration association. The differences in the duration of arrestin association would then determine whether receptors follow a ‘shallow’ intracellular pathway, to the ERC alone, or a ‘deeper’ pathway via the ERC to the TGN.

Our observations have been made in CHO-CCR5 cells, a model system in which studies of post-endocytic sorting are facilitated by the clearly distinct subcellular localizations of the ERC and the TGN [29]. As a next step it will be important to assess whether this phenomenon also occurs in primary cells expressing CCR5 such as T cells or macrophages. Further investigation of ligand-directed post-endocytic sorting of CCR5 should contribute to a deeper understanding of the inhibitory mechanisms of promising class of anti-HIV compounds, as well as providing new insights into the biology of GPCRs.
